# 2-Cyclo­hexyl-4-[(3,5-dimethyl­piperidin-1-yl)meth­yl]-5-methyl­phenol

**DOI:** 10.1107/S1600536809001305

**Published:** 2009-02-04

**Authors:** Gui-Yun Duan, Feng-Guang Guo, Cheng-Cai Xia

**Affiliations:** a College of Pharmaceutical Sciences, Taishan Medical University, Tai’an 271016, People’s Republic of China

## Abstract

The title compound, C_21_H_33_NO, crystallizes with three independent mol­ecules in the asymmetric unit. The cyclo­hexane and piperidine rings adopt chair conformations. The crystal packing is stabilized by inter­molecular O—H⋯N and C—H⋯O hydrogen bonds, and by weak π–π stacking inter­actions [centroid–centroid distance = 3.876 (2) Å].

## Related literature

For the biological activity of amidomethylphenol derivatives, see: Zhang *et al.* (1986 ▶). For bond-length data, see: Allen *et al.* (1987 ▶).
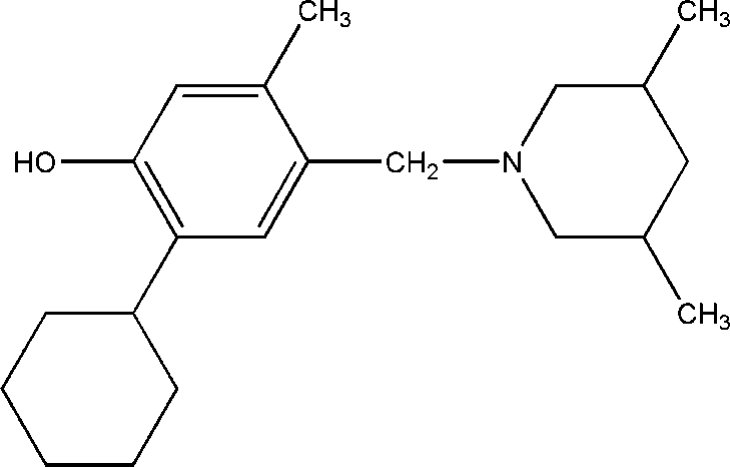


## Experimental

### 

#### Crystal data

C_21_H_33_NO
*M* *_r_* = 315.48
Triclinic, 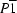

*a* = 12.0551 (7) Å
*b* = 16.5513 (10) Å
*c* = 16.7371 (10) Å
α = 78.969 (1)°
β = 69.354 (1)°
γ = 71.951 (1)°
*V* = 2958.6 (3) Å^3^
*Z* = 6
Mo *K*α radiation
μ = 0.06 mm^−1^
*T* = 273 (2) K
0.18 × 0.15 × 0.12 mm

#### Data collection

Bruker SMART CCD area-detector diffractometer
Absorption correction: none
15478 measured reflections
10326 independent reflections
6658 reflections with *I* > 2σ(*I*)
*R*_int_ = 0.016

#### Refinement

*R*[*F* ^2^ > 2σ(*F* ^2^)] = 0.067
*wR*(*F* ^2^) = 0.219
*S* = 1.03
10326 reflections
634 parameters
1296 restraints
H-atom parameters constrained
Δρ_max_ = 0.67 e Å^−3^
Δρ_min_ = −0.60 e Å^−3^

### 

Data collection: *SMART* (Bruker, 2001 ▶); cell refinement: *SAINT* (Bruker, 2001 ▶); data reduction: *SAINT*; program(s) used to solve structure: *SHELXTL* (Sheldrick, 2008 ▶); program(s) used to refine structure: *SHELXTL*; molecular graphics: *SHELXTL*; software used to prepare material for publication: *SHELXTL*.

## Supplementary Material

Crystal structure: contains datablocks I, global. DOI: 10.1107/S1600536809001305/hg2466sup1.cif

Structure factors: contains datablocks I. DOI: 10.1107/S1600536809001305/hg2466Isup2.hkl

Additional supplementary materials:  crystallographic information; 3D view; checkCIF report

## Figures and Tables

**Table 1:** Hydrogen-bond geometry (Å, °)

*D*—H⋯*A*	*D*—H	H⋯*A*	*D*⋯*A*	*D*—H⋯*A*
O1—H1⋯N1^i^	0.82	2.03	2.843 (3)	170
C13—H13*B*⋯O1^i^	0.96	2.52	3.335 (4)	143
O2—H2⋯N2^ii^	0.82	2.03	2.845 (3)	171
C34—H34*C*⋯O2^ii^	0.96	2.49	3.348 (4)	149
O3—H3⋯N3^iii^	0.82	2.02	2.831 (3)	174
C55—H55*C*⋯O3^iii^	0.96	2.58	3.404 (4)	145

Symmetry codes: (i) 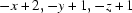; (ii) 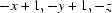; (iii) 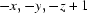.

**Fig. 1.**
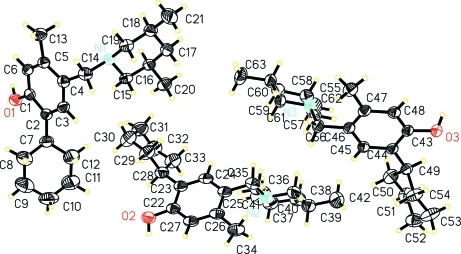
View of the title compound (I), with displacement ellipsoids drawn at the 35% probability level.

## supplementary crystallographic information

### Comment

4-amidomethyl-phenol derivatives have anti-inflammatory active [Zhang *et al.*, 1986]. The title compound is a derivative of it. In this paper, we report the crystal structure of the title compound.

The title compound, C_21_H_33_NO, crystallizes with three independent molecules in the asymmetric unit; all bond lengths are normal (Allen *et al.*, 1987). The cyclohexane (C7—C12; C28—C33; C49—C54) and piperidine ring (N1/C15—C19; N2/C36—C40; N3/C57—C61) adopt chair conformations. The relatively short distance of 3.876 (2)Å between the centroids of benzene ring C43—C48 [at (–*X*,-Y,1-*Z*)] indicates the presence of weak π-π interactions, which contribute to the stability of the crystal packing. The crystal packing is also stabilized by intermolecular O—H···N and C—H···O hydrogen bonds,

### Experimental

The title compound was prepared by reaction of 2-cyclohexyl-5-methylphenol (1.90 g, 0.01 mol) with 3,5-dimethylpiperidine(1.13 g, 0.01 mol) and 36% formaldehyde solution(1.00 g, 0.012 mol) in 10 mL of 95% alcohol under reflux for 3 h. After removing of the solvent, the residue was recrystallized from ethanol to afford 2.74 g of the title compound with yield of 87%. Crystals suitable for X-ray diffraction analysis were obtained by slow evaporation of a methanol solution at room temperature for two weeks.

### Refinement

All C atoms closer than 3.8Å are restrained with effective standard deviation 0.01Å to have the same U_ij_ components. All H atoms were placed in calculated positions, with C—H = 0.93–0.98Å, O—H = 0.82Å, and included in the final cycles of refinement using a riding model, with *U*_iso_(H) = 1.2 (1.5 for methyl and phenol)times *U*_eq_(C, O).

### Crystal data

**Table:**

C_21_H_33_NO	*Z* = 6
*M**_r_* = 315.48	*F*(000) = 1044
Triclinic, *P*1	*D*_x_ = 1.062 Mg m^−^^3^
Hall symbol: -P 1	Mo *K*α radiation, λ = 0.71073 Å
*a* = 12.0551 (7) Å	Cell parameters from 4912 reflections
*b* = 16.5513 (10) Å	θ = 2.6–24.6°
*c* = 16.7371 (10) Å	µ = 0.06 mm^−^^1^
α = 78.969 (1)°	*T* = 273 K
β = 69.354 (1)°	Block, colourless
γ = 71.951 (1)°	0.18 × 0.15 × 0.12 mm
*V* = 2958.6 (3) Å^3^	

### Data collection

**Table:**

Bruker SMART CCD area-detector diffractometer	6658 reflections with *I* > 2σ(*I*)
Radiation source: fine-focus sealed tube	*R*_int_ = 0.016
graphite	θ_max_ = 25.1°, θ_min_ = 1.3°
φ and ω scans	*h* = −13→14
15478 measured reflections	*k* = −19→19
10326 independent reflections	*l* = −19→15

### Refinement

**Table:**

Refinement on *F*^2^	Primary atom site location: structure-invariant direct methods
Least-squares matrix: full	Secondary atom site location: difference Fourier map
*R*[*F*^2^ > 2σ(*F*^2^)] = 0.067	Hydrogen site location: inferred from neighbouring sites
*wR*(*F*^2^) = 0.219	H-atom parameters constrained
*S* = 1.03	*w* = 1/[σ^2^(*F*_o_^2^) + (0.1179*P*)^2^ + 0.8741*P*] where *P* = (*F*_o_^2^ + 2*F*_c_^2^)/3
10326 reflections	(Δ/σ)_max_ = 0.003
634 parameters	Δρ_max_ = 0.67 e Å^−^^3^
1296 restraints	Δρ_min_ = −0.60 e Å^−^^3^

### Special details

**Table:**

Geometry. All e.s.d.'s (except the e.s.d. in the dihedral angle between two l.s. planes) are estimated using the full covariance matrix. The cell e.s.d.'s are taken into account individually in the estimation of e.s.d.'s in distances, angles and torsion angles; correlations between e.s.d.'s in cell parameters are only used when they are defined by crystal symmetry. An approximate (isotropic) treatment of cell e.s.d.'s is used for estimating e.s.d.'s involving l.s. planes.
Refinement. Refinement of *F*^2^ against ALL reflections. The weighted *R*-factor *wR* and goodness of fit *S* are based on *F*^2^, conventional *R*-factors *R* are based on *F*, with *F* set to zero for negative *F*^2^. The threshold expression of *F*^2^ > σ(*F*^2^) is used only for calculating *R*-factors(gt) *etc*. and is not relevant to the choice of reflections for refinement. *R*-factors based on *F*^2^ are statistically about twice as large as those based on *F*, and *R*- factors based on ALL data will be even larger.

### Fractional atomic coordinates and isotropic or equivalent isotropic displacement parameters (Å^2^)

**Table:**

	*x*	*y*	*z*	*U*_iso_*/*U*_eq_	
O1	1.10328 (19)	0.64137 (14)	0.37255 (14)	0.0619 (5)	
H1	1.1584	0.6115	0.3915	0.093*	
O2	0.6324 (2)	0.59539 (14)	0.06614 (15)	0.0715 (6)	
H2	0.6097	0.6263	0.0272	0.107*	
O3	−0.21541 (19)	−0.00005 (13)	0.43573 (14)	0.0584 (5)	
H3	−0.2175	−0.0355	0.4775	0.088*	
N1	0.6856 (2)	0.46313 (14)	0.58429 (14)	0.0487 (5)	
N2	0.4452 (2)	0.28225 (16)	0.06114 (17)	0.0611 (7)	
N3	0.2293 (2)	0.13009 (14)	0.42672 (15)	0.0494 (6)	
C1	0.9930 (3)	0.62786 (17)	0.42522 (19)	0.0510 (7)	
C2	0.8988 (3)	0.64123 (17)	0.38983 (18)	0.0519 (7)	
C3	0.7938 (3)	0.61861 (17)	0.44360 (19)	0.0532 (7)	
H3A	0.7312	0.6246	0.4210	0.064*	
C4	0.7757 (3)	0.58758 (17)	0.52915 (18)	0.0501 (6)	
C5	0.8661 (3)	0.58369 (16)	0.56485 (18)	0.0495 (6)	
C6	0.9730 (3)	0.60285 (16)	0.51190 (18)	0.0503 (6)	
H6	1.0344	0.5989	0.5351	0.060*	
C7	0.9133 (3)	0.6773 (2)	0.2981 (2)	0.0706 (8)	
H7	0.9991	0.6532	0.2652	0.085*	
C8	0.8928 (6)	0.7691 (3)	0.2858 (3)	0.1133 (13)	
H8A	0.9491	0.7843	0.3060	0.136*	
H8B	0.8098	0.7953	0.3201	0.136*	
C9	0.9104 (7)	0.8048 (4)	0.1929 (4)	0.1432 (17)	
H9A	0.8829	0.8665	0.1910	0.172*	
H9B	0.9977	0.7896	0.1618	0.172*	
C10	0.8498 (6)	0.7776 (4)	0.1498 (3)	0.1223 (15)	
H10A	0.8817	0.7936	0.0889	0.147*	
H10B	0.7631	0.8072	0.1688	0.147*	
C11	0.8626 (6)	0.6886 (4)	0.1629 (3)	0.1261 (15)	
H11A	0.9454	0.6594	0.1309	0.151*	
H11B	0.8060	0.6758	0.1409	0.151*	
C12	0.8373 (5)	0.6555 (3)	0.2571 (3)	0.1059 (12)	
H12A	0.7512	0.6790	0.2876	0.127*	
H12B	0.8526	0.5939	0.2622	0.127*	
C13	0.8491 (3)	0.5612 (2)	0.65912 (19)	0.0664 (8)	
H13A	0.9223	0.5603	0.6704	0.100*	
H13B	0.8335	0.5059	0.6759	0.100*	
H13C	0.7808	0.6029	0.6912	0.100*	
C14	0.6631 (3)	0.55707 (18)	0.5806 (2)	0.0574 (7)	
H14A	0.6354	0.5735	0.6386	0.069*	
H14B	0.5980	0.5851	0.5555	0.069*	
C15	0.6948 (3)	0.43837 (18)	0.50216 (18)	0.0519 (7)	
H15A	0.7600	0.4581	0.4570	0.062*	
H15B	0.6185	0.4662	0.4895	0.062*	
C16	0.7207 (3)	0.34242 (18)	0.50235 (18)	0.0534 (7)	
H16	0.7994	0.3155	0.5133	0.064*	
C17	0.6217 (3)	0.3107 (2)	0.5750 (2)	0.0617 (8)	
H17A	0.5448	0.3314	0.5620	0.074*	
H17B	0.6438	0.2488	0.5794	0.074*	
C18	0.6036 (3)	0.3397 (2)	0.66034 (19)	0.0605 (7)	
H18	0.6782	0.3118	0.6764	0.073*	
C19	0.5851 (3)	0.4355 (2)	0.65256 (19)	0.0577 (7)	
H19A	0.5083	0.4641	0.6409	0.069*	
H19B	0.5792	0.4522	0.7067	0.069*	
C20	0.7321 (3)	0.3187 (2)	0.4159 (2)	0.0706 (9)	
H20A	0.7473	0.2580	0.4181	0.106*	
H20B	0.7990	0.3367	0.3724	0.106*	
H20C	0.6571	0.3464	0.4027	0.106*	
C21	0.4957 (4)	0.3140 (3)	0.7307 (3)	0.0894 (11)	
H21A	0.4214	0.3411	0.7164	0.134*	
H21B	0.4882	0.3316	0.7842	0.134*	
H21C	0.5097	0.2532	0.7357	0.134*	
C22	0.5783 (3)	0.5293 (2)	0.09074 (18)	0.0602 (7)	
C23	0.6489 (3)	0.4483 (2)	0.11016 (19)	0.0619 (8)	
C24	0.5923 (3)	0.3825 (2)	0.1302 (2)	0.0663 (8)	
H24	0.6368	0.3281	0.1446	0.080*	
C25	0.4732 (3)	0.3923 (2)	0.13011 (19)	0.0630 (8)	
C26	0.4017 (3)	0.4754 (2)	0.11550 (18)	0.0620 (8)	
C27	0.4568 (3)	0.5415 (2)	0.09701 (18)	0.0622 (8)	
H27	0.4100	0.5968	0.0884	0.075*	
C28	0.7785 (3)	0.4360 (2)	0.1079 (2)	0.0700 (8)	
H28	0.8096	0.4805	0.0662	0.084*	
C29	0.7838 (4)	0.4489 (3)	0.1931 (3)	0.0876 (10)	
H29A	0.7468	0.4089	0.2369	0.105*	
H29B	0.7356	0.5061	0.2077	0.105*	
C30	0.9135 (4)	0.4367 (3)	0.1936 (3)	0.1053 (12)	
H30A	0.9447	0.4834	0.1580	0.126*	
H30B	0.9112	0.4391	0.2516	0.126*	
C31	0.9995 (4)	0.3547 (3)	0.1620 (3)	0.1037 (12)	
H31A	1.0823	0.3537	0.1575	0.124*	
H31B	0.9771	0.3077	0.2029	0.124*	
C32	0.9964 (4)	0.3432 (3)	0.0769 (3)	0.1060 (13)	
H32A	1.0488	0.2876	0.0599	0.127*	
H32B	1.0284	0.3861	0.0345	0.127*	
C33	0.8665 (4)	0.3505 (3)	0.0791 (3)	0.0946 (11)	
H33A	0.8371	0.3046	0.1180	0.113*	
H33B	0.8675	0.3442	0.0224	0.113*	
C34	0.2702 (3)	0.4943 (2)	0.1181 (2)	0.0763 (10)	
H34A	0.2377	0.5548	0.1086	0.114*	
H34B	0.2228	0.4737	0.1732	0.114*	
H34C	0.2664	0.4664	0.0743	0.114*	
C35	0.4258 (4)	0.3152 (2)	0.1425 (2)	0.0731 (9)	
H35A	0.4665	0.2703	0.1773	0.088*	
H35B	0.3386	0.3299	0.1738	0.088*	
C36	0.5750 (3)	0.2413 (2)	0.0207 (2)	0.0756 (9)	
H36A	0.6034	0.1941	0.0592	0.091*	
H36B	0.6228	0.2820	0.0104	0.091*	
C37	0.5955 (4)	0.2089 (3)	−0.0631 (3)	0.0869 (11)	
H37	0.5665	0.2577	−0.1009	0.104*	
C38	0.5210 (4)	0.1466 (3)	−0.0487 (3)	0.0957 (12)	
H38A	0.5533	0.0954	−0.0158	0.115*	
H38B	0.5290	0.1305	−0.1037	0.115*	
C39	0.3860 (4)	0.1833 (2)	−0.0015 (3)	0.0838 (10)	
H39	0.3530	0.2305	−0.0392	0.101*	
C40	0.3732 (4)	0.2195 (2)	0.0780 (3)	0.0780 (9)	
H40A	0.2872	0.2468	0.1050	0.094*	
H40B	0.3998	0.1731	0.1180	0.094*	
C41	0.7318 (5)	0.1706 (4)	−0.1068 (4)	0.147 (2)	
H41A	0.7637	0.1247	−0.0696	0.221*	
H41B	0.7750	0.2138	−0.1192	0.221*	
H41C	0.7425	0.1493	−0.1593	0.221*	
C42	0.3106 (5)	0.1188 (3)	0.0200 (4)	0.1225 (17)	
H42A	0.3324	0.0764	0.0636	0.184*	
H42B	0.3272	0.0919	−0.0305	0.184*	
H42C	0.2248	0.1475	0.0403	0.184*	
C43	−0.1430 (2)	0.05018 (16)	0.43318 (19)	0.0480 (6)	
C44	−0.0715 (3)	0.07848 (16)	0.35358 (18)	0.0480 (6)	
C45	0.0061 (3)	0.12507 (16)	0.35401 (19)	0.0499 (6)	
H45	0.0543	0.1450	0.3015	0.060*	
C46	0.0160 (3)	0.14376 (16)	0.42838 (19)	0.0496 (6)	
C47	−0.0631 (3)	0.11979 (16)	0.50753 (19)	0.0507 (7)	
C48	−0.1412 (3)	0.07321 (17)	0.50803 (19)	0.0506 (6)	
H48	−0.1940	0.0569	0.5602	0.061*	
C49	−0.0817 (3)	0.05966 (18)	0.27171 (19)	0.0533 (7)	
H49	−0.0995	0.0039	0.2827	0.064*	
C50	0.0341 (3)	0.0550 (3)	0.1946 (2)	0.0772 (9)	
H50A	0.1020	0.0114	0.2080	0.093*	
H50B	0.0548	0.1092	0.1825	0.093*	
C51	0.0166 (4)	0.0342 (3)	0.1154 (2)	0.0842 (10)	
H51A	0.0901	0.0346	0.0668	0.101*	
H51B	0.0044	−0.0226	0.1255	0.101*	
C52	−0.0920 (4)	0.0976 (3)	0.0949 (2)	0.0906 (11)	
H52A	−0.0749	0.1530	0.0773	0.109*	
H52B	−0.1049	0.0800	0.0476	0.109*	
C53	−0.2063 (4)	0.1049 (3)	0.1706 (3)	0.0969 (12)	
H53A	−0.2297	0.0517	0.1832	0.116*	
H53B	−0.2726	0.1496	0.1565	0.116*	
C54	−0.1892 (3)	0.1251 (2)	0.2497 (2)	0.0748 (9)	
H54A	−0.1751	0.1813	0.2395	0.090*	
H54B	−0.2636	0.1259	0.2979	0.090*	
C55	−0.0657 (3)	0.14181 (19)	0.5918 (2)	0.0628 (8)	
H55A	−0.1209	0.1155	0.6382	0.094*	
H55B	−0.0932	0.2026	0.5938	0.094*	
H55C	0.0154	0.1213	0.5971	0.094*	
C56	0.1100 (3)	0.18862 (17)	0.4224 (2)	0.0565 (7)	
H56A	0.1239	0.2248	0.3689	0.068*	
H56B	0.0772	0.2252	0.4688	0.068*	
C57	0.2979 (3)	0.08899 (19)	0.3469 (2)	0.0596 (7)	
H57A	0.3113	0.1326	0.2994	0.072*	
H57B	0.2492	0.0572	0.3370	0.072*	
C58	0.4213 (3)	0.0288 (2)	0.3492 (2)	0.0686 (8)	
H58	0.4057	−0.0158	0.3963	0.082*	
C59	0.4963 (3)	0.0770 (2)	0.3680 (2)	0.0721 (9)	
H59A	0.5712	0.0371	0.3751	0.087*	
H59B	0.5193	0.1179	0.3195	0.087*	
C60	0.4267 (3)	0.1235 (2)	0.4478 (2)	0.0648 (8)	
H60	0.4121	0.0807	0.4969	0.078*	
C61	0.3019 (3)	0.17908 (19)	0.4420 (2)	0.0588 (7)	
H61A	0.2561	0.2058	0.4950	0.071*	
H61B	0.3144	0.2240	0.3958	0.071*	
C62	0.4879 (4)	−0.0142 (3)	0.2659 (3)	0.1078 (14)	
H62A	0.5048	0.0283	0.2187	0.162*	
H62B	0.4370	−0.0441	0.2571	0.162*	
H62C	0.5637	−0.0539	0.2694	0.162*	
C63	0.4964 (4)	0.1775 (3)	0.4643 (3)	0.0931 (12)	
H63A	0.5750	0.1422	0.4675	0.140*	
H63B	0.4503	0.2024	0.5175	0.140*	
H63C	0.5079	0.2219	0.4185	0.140*	

### Atomic displacement parameters (Å^2^)

**Table:**

	*U*^11^	*U*^22^	*U*^33^	*U*^12^	*U*^13^	*U*^23^
O1	0.0532 (12)	0.0626 (13)	0.0691 (14)	−0.0152 (10)	−0.0237 (11)	0.0043 (10)
O2	0.0892 (17)	0.0569 (13)	0.0712 (15)	−0.0115 (12)	−0.0379 (13)	−0.0005 (11)
O3	0.0615 (13)	0.0554 (12)	0.0710 (14)	−0.0242 (10)	−0.0341 (11)	0.0048 (10)
N1	0.0448 (13)	0.0522 (13)	0.0446 (13)	−0.0079 (10)	−0.0132 (10)	−0.0036 (10)
N2	0.0621 (16)	0.0525 (14)	0.0649 (16)	−0.0071 (12)	−0.0263 (13)	0.0020 (12)
N3	0.0512 (13)	0.0420 (12)	0.0612 (15)	−0.0105 (10)	−0.0254 (12)	−0.0068 (10)
C1	0.0568 (16)	0.0406 (13)	0.0564 (16)	−0.0098 (12)	−0.0206 (13)	−0.0061 (11)
C2	0.0574 (16)	0.0460 (14)	0.0536 (15)	−0.0100 (12)	−0.0228 (13)	−0.0031 (12)
C3	0.0551 (15)	0.0501 (14)	0.0571 (16)	−0.0066 (12)	−0.0272 (13)	−0.0051 (12)
C4	0.0505 (15)	0.0404 (13)	0.0536 (15)	−0.0025 (11)	−0.0146 (12)	−0.0100 (11)
C5	0.0571 (16)	0.0386 (13)	0.0494 (15)	−0.0014 (12)	−0.0194 (13)	−0.0095 (11)
C6	0.0562 (16)	0.0416 (13)	0.0575 (16)	−0.0043 (12)	−0.0280 (13)	−0.0093 (12)
C7	0.077 (2)	0.0777 (19)	0.0601 (17)	−0.0229 (16)	−0.0304 (15)	0.0085 (15)
C8	0.173 (3)	0.089 (2)	0.092 (2)	−0.048 (2)	−0.061 (2)	0.016 (2)
C9	0.208 (4)	0.119 (3)	0.112 (3)	−0.064 (3)	−0.067 (3)	0.035 (3)
C10	0.148 (3)	0.135 (3)	0.085 (3)	−0.034 (3)	−0.063 (3)	0.032 (3)
C11	0.178 (4)	0.137 (3)	0.077 (2)	−0.049 (3)	−0.057 (3)	0.004 (2)
C12	0.146 (3)	0.108 (2)	0.078 (2)	−0.045 (2)	−0.049 (2)	0.0006 (19)
C13	0.081 (2)	0.0647 (19)	0.0506 (17)	−0.0082 (16)	−0.0224 (16)	−0.0126 (14)
C14	0.0505 (16)	0.0531 (15)	0.0585 (16)	−0.0005 (12)	−0.0147 (13)	−0.0091 (13)
C15	0.0454 (14)	0.0581 (15)	0.0487 (15)	−0.0057 (12)	−0.0197 (12)	−0.0005 (12)
C16	0.0506 (16)	0.0551 (16)	0.0542 (16)	−0.0066 (13)	−0.0226 (13)	−0.0045 (12)
C17	0.0588 (17)	0.0635 (17)	0.0653 (18)	−0.0193 (14)	−0.0229 (15)	−0.0001 (14)
C18	0.0585 (17)	0.0688 (18)	0.0538 (17)	−0.0207 (14)	−0.0192 (14)	0.0047 (14)
C19	0.0490 (16)	0.0680 (17)	0.0502 (16)	−0.0107 (13)	−0.0136 (13)	−0.0037 (13)
C20	0.074 (2)	0.076 (2)	0.063 (2)	−0.0099 (17)	−0.0256 (17)	−0.0169 (16)
C21	0.092 (3)	0.097 (3)	0.074 (2)	−0.040 (2)	−0.013 (2)	0.005 (2)
C22	0.0768 (19)	0.0560 (16)	0.0434 (15)	−0.0065 (14)	−0.0221 (14)	−0.0067 (12)
C23	0.0769 (19)	0.0579 (16)	0.0490 (15)	−0.0081 (15)	−0.0262 (14)	−0.0046 (13)
C24	0.081 (2)	0.0585 (16)	0.0548 (16)	−0.0058 (15)	−0.0313 (15)	0.0031 (13)
C25	0.0766 (19)	0.0645 (17)	0.0440 (15)	−0.0138 (15)	−0.0224 (14)	0.0027 (13)
C26	0.0727 (19)	0.0670 (18)	0.0366 (14)	−0.0059 (15)	−0.0168 (13)	−0.0042 (13)
C27	0.0743 (19)	0.0570 (16)	0.0446 (15)	0.0020 (15)	−0.0213 (14)	−0.0073 (13)
C28	0.0757 (19)	0.0617 (17)	0.0690 (18)	−0.0042 (15)	−0.0324 (16)	−0.0025 (14)
C29	0.087 (2)	0.095 (2)	0.087 (2)	−0.0045 (19)	−0.0407 (18)	−0.0270 (18)
C30	0.101 (3)	0.114 (3)	0.114 (3)	−0.010 (2)	−0.053 (2)	−0.032 (2)
C31	0.089 (3)	0.102 (3)	0.119 (3)	−0.003 (2)	−0.052 (2)	−0.008 (2)
C32	0.086 (3)	0.106 (3)	0.113 (3)	0.003 (2)	−0.032 (2)	−0.027 (2)
C33	0.093 (2)	0.091 (2)	0.094 (2)	0.0031 (19)	−0.035 (2)	−0.0286 (19)
C34	0.070 (2)	0.089 (2)	0.0533 (19)	−0.0045 (18)	−0.0156 (16)	−0.0017 (17)
C35	0.084 (2)	0.0686 (18)	0.0595 (18)	−0.0156 (16)	−0.0249 (16)	0.0062 (15)
C36	0.070 (2)	0.0692 (19)	0.083 (2)	0.0011 (16)	−0.0373 (17)	−0.0032 (16)
C37	0.079 (2)	0.078 (2)	0.093 (2)	0.0077 (18)	−0.0316 (19)	−0.0208 (19)
C38	0.105 (3)	0.072 (2)	0.108 (3)	0.003 (2)	−0.047 (2)	−0.019 (2)
C39	0.089 (2)	0.0624 (19)	0.105 (3)	−0.0099 (18)	−0.048 (2)	−0.0024 (18)
C40	0.078 (2)	0.0641 (19)	0.090 (2)	−0.0140 (16)	−0.0344 (18)	0.0059 (17)
C41	0.096 (4)	0.152 (5)	0.155 (5)	0.024 (3)	−0.015 (3)	−0.067 (4)
C42	0.142 (4)	0.089 (3)	0.162 (5)	−0.040 (3)	−0.077 (4)	0.000 (3)
C43	0.0455 (14)	0.0388 (13)	0.0641 (16)	−0.0054 (11)	−0.0290 (13)	−0.0019 (12)
C44	0.0506 (15)	0.0379 (13)	0.0588 (16)	−0.0049 (11)	−0.0288 (13)	−0.0010 (11)
C45	0.0507 (15)	0.0407 (13)	0.0606 (16)	−0.0087 (11)	−0.0253 (12)	−0.0003 (11)
C46	0.0511 (15)	0.0352 (12)	0.0676 (17)	−0.0045 (11)	−0.0304 (13)	−0.0046 (12)
C47	0.0546 (16)	0.0375 (13)	0.0642 (17)	0.0002 (12)	−0.0332 (14)	−0.0073 (12)
C48	0.0488 (15)	0.0424 (14)	0.0599 (16)	−0.0047 (12)	−0.0238 (13)	−0.0020 (12)
C49	0.0592 (16)	0.0489 (14)	0.0597 (16)	−0.0138 (12)	−0.0302 (13)	−0.0019 (12)
C50	0.073 (2)	0.086 (2)	0.074 (2)	−0.0092 (17)	−0.0258 (17)	−0.0241 (17)
C51	0.098 (3)	0.085 (2)	0.072 (2)	−0.020 (2)	−0.0243 (19)	−0.0259 (18)
C52	0.118 (3)	0.086 (2)	0.068 (2)	−0.012 (2)	−0.042 (2)	−0.0094 (18)
C53	0.095 (3)	0.118 (3)	0.081 (2)	0.004 (2)	−0.057 (2)	−0.016 (2)
C54	0.0678 (19)	0.086 (2)	0.0661 (18)	0.0032 (16)	−0.0348 (16)	−0.0106 (16)
C55	0.067 (2)	0.0528 (17)	0.071 (2)	−0.0017 (14)	−0.0328 (16)	−0.0148 (14)
C56	0.0582 (16)	0.0414 (14)	0.0776 (18)	−0.0100 (12)	−0.0335 (14)	−0.0054 (13)
C57	0.0646 (17)	0.0523 (15)	0.0641 (17)	−0.0143 (13)	−0.0231 (14)	−0.0064 (13)
C58	0.0635 (19)	0.0571 (17)	0.078 (2)	−0.0057 (15)	−0.0186 (16)	−0.0125 (15)
C59	0.0561 (18)	0.0654 (19)	0.090 (2)	−0.0106 (15)	−0.0238 (17)	−0.0035 (17)
C60	0.0588 (18)	0.0593 (17)	0.085 (2)	−0.0185 (14)	−0.0322 (16)	−0.0022 (15)
C61	0.0570 (17)	0.0498 (15)	0.0788 (19)	−0.0168 (13)	−0.0296 (15)	−0.0069 (14)
C62	0.092 (3)	0.106 (3)	0.109 (3)	−0.001 (2)	−0.015 (3)	−0.047 (3)
C63	0.069 (2)	0.094 (3)	0.137 (4)	−0.025 (2)	−0.050 (2)	−0.020 (2)

### Geometric parameters (Å, °)

**Table:**

O1—C1	1.370 (3)	C30—H30B	0.9700
O1—H1	0.8200	C31—C32	1.488 (6)
O2—C22	1.367 (4)	C31—H31A	0.9700
O2—H2	0.8200	C31—H31B	0.9700
O3—C43	1.366 (3)	C32—C33	1.521 (6)
O3—H3	0.8200	C32—H32A	0.9700
N1—C15	1.466 (3)	C32—H32B	0.9700
N1—C19	1.467 (4)	C33—H33A	0.9700
N1—C14	1.486 (4)	C33—H33B	0.9700
N2—C36	1.462 (4)	C34—H34A	0.9600
N2—C40	1.478 (4)	C34—H34B	0.9600
N2—C35	1.480 (4)	C34—H34C	0.9600
N3—C57	1.467 (4)	C35—H35A	0.9700
N3—C61	1.471 (3)	C35—H35B	0.9700
N3—C56	1.482 (4)	C36—C37	1.509 (5)
C1—C6	1.386 (4)	C36—H36A	0.9700
C1—C2	1.399 (4)	C36—H36B	0.9700
C2—C3	1.383 (4)	C37—C38	1.502 (6)
C2—C7	1.507 (4)	C37—C41	1.523 (6)
C3—C4	1.389 (4)	C37—H37	0.9800
C3—H3A	0.9300	C38—C39	1.519 (6)
C4—C5	1.397 (4)	C38—H38A	0.9700
C4—C14	1.507 (4)	C38—H38B	0.9700
C5—C6	1.374 (4)	C39—C40	1.503 (5)
C5—C13	1.505 (4)	C39—C42	1.522 (6)
C6—H6	0.9300	C39—H39	0.9800
C7—C8	1.447 (5)	C40—H40A	0.9700
C7—C12	1.478 (5)	C40—H40B	0.9700
C7—H7	0.9800	C41—H41A	0.9600
C8—C9	1.518 (7)	C41—H41B	0.9600
C8—H8A	0.9700	C41—H41C	0.9600
C8—H8B	0.9700	C42—H42A	0.9600
C9—C10	1.398 (7)	C42—H42B	0.9600
C9—H9A	0.9700	C42—H42C	0.9600
C9—H9B	0.9700	C43—C48	1.386 (4)
C10—C11	1.415 (7)	C43—C44	1.392 (4)
C10—H10A	0.9700	C44—C45	1.387 (4)
C10—H10B	0.9700	C44—C49	1.515 (4)
C11—C12	1.521 (6)	C45—C46	1.391 (4)
C11—H11A	0.9700	C45—H45	0.9300
C11—H11B	0.9700	C46—C47	1.402 (4)
C12—H12A	0.9700	C46—C56	1.503 (4)
C12—H12B	0.9700	C47—C48	1.386 (4)
C13—H13A	0.9600	C47—C55	1.511 (4)
C13—H13B	0.9600	C48—H48	0.9300
C13—H13C	0.9600	C49—C54	1.517 (4)
C14—H14A	0.9700	C49—C50	1.524 (5)
C14—H14B	0.9700	C49—H49	0.9800
C15—C16	1.520 (4)	C50—C51	1.529 (5)
C15—H15A	0.9700	C50—H50A	0.9700
C15—H15B	0.9700	C50—H50B	0.9700
C16—C17	1.517 (4)	C51—C52	1.503 (6)
C16—C20	1.520 (4)	C51—H51A	0.9700
C16—H16	0.9800	C51—H51B	0.9700
C17—C18	1.515 (4)	C52—C53	1.498 (6)
C17—H17A	0.9700	C52—H52A	0.9700
C17—H17B	0.9700	C52—H52B	0.9700
C18—C19	1.517 (4)	C53—C54	1.520 (5)
C18—C21	1.526 (5)	C53—H53A	0.9700
C18—H18	0.9800	C53—H53B	0.9700
C19—H19A	0.9700	C54—H54A	0.9700
C19—H19B	0.9700	C54—H54B	0.9700
C20—H20A	0.9600	C55—H55A	0.9600
C20—H20B	0.9600	C55—H55B	0.9600
C20—H20C	0.9600	C55—H55C	0.9600
C21—H21A	0.9600	C56—H56A	0.9700
C21—H21B	0.9600	C56—H56B	0.9700
C21—H21C	0.9600	C57—C58	1.522 (4)
C22—C27	1.383 (5)	C57—H57A	0.9700
C22—C23	1.397 (4)	C57—H57B	0.9700
C23—C24	1.386 (5)	C58—C59	1.515 (5)
C23—C28	1.500 (5)	C58—C62	1.527 (5)
C24—C25	1.394 (5)	C58—H58	0.9800
C24—H24	0.9300	C59—C60	1.512 (5)
C25—C26	1.407 (4)	C59—H59A	0.9700
C25—C35	1.505 (5)	C59—H59B	0.9700
C26—C27	1.383 (5)	C60—C63	1.518 (4)
C26—C34	1.503 (5)	C60—C61	1.526 (4)
C27—H27	0.9300	C60—H60	0.9800
C28—C29	1.509 (5)	C61—H61A	0.9700
C28—C33	1.527 (5)	C61—H61B	0.9700
C28—H28	0.9800	C62—H62A	0.9600
C29—C30	1.516 (6)	C62—H62B	0.9600
C29—H29A	0.9700	C62—H62C	0.9600
C29—H29B	0.9700	C63—H63A	0.9600
C30—C31	1.488 (6)	C63—H63B	0.9600
C30—H30A	0.9700	C63—H63C	0.9600
			
C1—O1—H1	109.5	C32—C33—C28	111.8 (4)
C22—O2—H2	109.5	C32—C33—H33A	109.2
C43—O3—H3	109.5	C28—C33—H33A	109.2
C15—N1—C19	109.5 (2)	C32—C33—H33B	109.2
C15—N1—C14	110.7 (2)	C28—C33—H33B	109.2
C19—N1—C14	109.7 (2)	H33A—C33—H33B	107.9
C36—N2—C40	109.1 (3)	C26—C34—H34A	109.5
C36—N2—C35	111.3 (3)	C26—C34—H34B	109.5
C40—N2—C35	109.5 (3)	H34A—C34—H34B	109.5
C57—N3—C61	109.7 (2)	C26—C34—H34C	109.5
C57—N3—C56	111.1 (2)	H34A—C34—H34C	109.5
C61—N3—C56	108.9 (2)	H34B—C34—H34C	109.5
O1—C1—C6	121.5 (3)	N2—C35—C25	113.4 (3)
O1—C1—C2	118.4 (3)	N2—C35—H35A	108.9
C6—C1—C2	120.1 (3)	C25—C35—H35A	108.9
C3—C2—C1	116.1 (3)	N2—C35—H35B	108.9
C3—C2—C7	122.7 (3)	C25—C35—H35B	108.9
C1—C2—C7	121.2 (3)	H35A—C35—H35B	107.7
C2—C3—C4	124.4 (3)	N2—C36—C37	111.4 (3)
C2—C3—H3A	117.8	N2—C36—H36A	109.3
C4—C3—H3A	117.8	C37—C36—H36A	109.3
C3—C4—C5	118.0 (3)	N2—C36—H36B	109.3
C3—C4—C14	120.6 (3)	C37—C36—H36B	109.3
C5—C4—C14	121.4 (3)	H36A—C36—H36B	108.0
C6—C5—C4	118.5 (3)	C38—C37—C36	110.2 (4)
C6—C5—C13	119.2 (3)	C38—C37—C41	112.1 (4)
C4—C5—C13	122.3 (3)	C36—C37—C41	110.9 (4)
C5—C6—C1	122.5 (3)	C38—C37—H37	107.8
C5—C6—H6	118.7	C36—C37—H37	107.8
C1—C6—H6	118.7	C41—C37—H37	107.8
C8—C7—C12	107.5 (4)	C37—C38—C39	112.2 (3)
C8—C7—C2	113.5 (3)	C37—C38—H38A	109.2
C12—C7—C2	115.8 (3)	C39—C38—H38A	109.2
C8—C7—H7	106.5	C37—C38—H38B	109.2
C12—C7—H7	106.5	C39—C38—H38B	109.2
C2—C7—H7	106.5	H38A—C38—H38B	107.9
C7—C8—C9	113.2 (4)	C40—C39—C38	109.8 (3)
C7—C8—H8A	108.9	C40—C39—C42	110.9 (4)
C9—C8—H8A	108.9	C38—C39—C42	113.0 (4)
C7—C8—H8B	108.9	C40—C39—H39	107.7
C9—C8—H8B	108.9	C38—C39—H39	107.7
H8A—C8—H8B	107.8	C42—C39—H39	107.7
C10—C9—C8	116.2 (5)	N2—C40—C39	113.3 (3)
C10—C9—H9A	108.2	N2—C40—H40A	108.9
C8—C9—H9A	108.2	C39—C40—H40A	108.9
C10—C9—H9B	108.2	N2—C40—H40B	108.9
C8—C9—H9B	108.2	C39—C40—H40B	108.9
H9A—C9—H9B	107.4	H40A—C40—H40B	107.7
C9—C10—C11	113.6 (5)	C37—C41—H41A	109.5
C9—C10—H10A	108.9	C37—C41—H41B	109.5
C11—C10—H10A	108.9	H41A—C41—H41B	109.5
C9—C10—H10B	108.9	C37—C41—H41C	109.5
C11—C10—H10B	108.9	H41A—C41—H41C	109.5
H10A—C10—H10B	107.7	H41B—C41—H41C	109.5
C10—C11—C12	112.2 (4)	C39—C42—H42A	109.5
C10—C11—H11A	109.2	C39—C42—H42B	109.5
C12—C11—H11A	109.2	H42A—C42—H42B	109.5
C10—C11—H11B	109.2	C39—C42—H42C	109.5
C12—C11—H11B	109.2	H42A—C42—H42C	109.5
H11A—C11—H11B	107.9	H42B—C42—H42C	109.5
C7—C12—C11	113.2 (4)	O3—C43—C48	120.9 (3)
C7—C12—H12A	108.9	O3—C43—C44	118.5 (2)
C11—C12—H12A	108.9	C48—C43—C44	120.6 (2)
C7—C12—H12B	108.9	C45—C44—C43	116.6 (3)
C11—C12—H12B	108.9	C45—C44—C49	122.9 (3)
H12A—C12—H12B	107.7	C43—C44—C49	120.5 (2)
C5—C13—H13A	109.5	C44—C45—C46	123.8 (3)
C5—C13—H13B	109.5	C44—C45—H45	118.1
H13A—C13—H13B	109.5	C46—C45—H45	118.1
C5—C13—H13C	109.5	C45—C46—C47	118.4 (2)
H13A—C13—H13C	109.5	C45—C46—C56	119.9 (3)
H13B—C13—H13C	109.5	C47—C46—C56	121.7 (3)
N1—C14—C4	112.9 (2)	C48—C47—C46	118.2 (3)
N1—C14—H14A	109.0	C48—C47—C55	119.0 (3)
C4—C14—H14A	109.0	C46—C47—C55	122.8 (3)
N1—C14—H14B	109.0	C47—C48—C43	122.1 (3)
C4—C14—H14B	109.0	C47—C48—H48	119.0
H14A—C14—H14B	107.8	C43—C48—H48	119.0
N1—C15—C16	112.5 (2)	C44—C49—C54	110.1 (2)
N1—C15—H15A	109.1	C44—C49—C50	114.7 (2)
C16—C15—H15A	109.1	C54—C49—C50	109.6 (3)
N1—C15—H15B	109.1	C44—C49—H49	107.4
C16—C15—H15B	109.1	C54—C49—H49	107.4
H15A—C15—H15B	107.8	C50—C49—H49	107.4
C17—C16—C20	112.2 (3)	C49—C50—C51	111.3 (3)
C17—C16—C15	109.4 (2)	C49—C50—H50A	109.4
C20—C16—C15	111.2 (2)	C51—C50—H50A	109.4
C17—C16—H16	108.0	C49—C50—H50B	109.4
C20—C16—H16	108.0	C51—C50—H50B	109.4
C15—C16—H16	108.0	H50A—C50—H50B	108.0
C18—C17—C16	112.4 (2)	C52—C51—C50	111.3 (3)
C18—C17—H17A	109.1	C52—C51—H51A	109.4
C16—C17—H17A	109.1	C50—C51—H51A	109.4
C18—C17—H17B	109.1	C52—C51—H51B	109.4
C16—C17—H17B	109.1	C50—C51—H51B	109.4
H17A—C17—H17B	107.8	H51A—C51—H51B	108.0
C17—C18—C19	110.4 (2)	C53—C52—C51	111.6 (3)
C17—C18—C21	111.4 (3)	C53—C52—H52A	109.3
C19—C18—C21	110.5 (3)	C51—C52—H52A	109.3
C17—C18—H18	108.1	C53—C52—H52B	109.3
C19—C18—H18	108.1	C51—C52—H52B	109.3
C21—C18—H18	108.1	H52A—C52—H52B	108.0
N1—C19—C18	112.3 (2)	C52—C53—C54	112.2 (3)
N1—C19—H19A	109.1	C52—C53—H53A	109.2
C18—C19—H19A	109.1	C54—C53—H53A	109.2
N1—C19—H19B	109.1	C52—C53—H53B	109.2
C18—C19—H19B	109.1	C54—C53—H53B	109.2
H19A—C19—H19B	107.9	H53A—C53—H53B	107.9
C16—C20—H20A	109.5	C49—C54—C53	111.3 (3)
C16—C20—H20B	109.5	C49—C54—H54A	109.4
H20A—C20—H20B	109.5	C53—C54—H54A	109.4
C16—C20—H20C	109.5	C49—C54—H54B	109.4
H20A—C20—H20C	109.5	C53—C54—H54B	109.4
H20B—C20—H20C	109.5	H54A—C54—H54B	108.0
C18—C21—H21A	109.5	C47—C55—H55A	109.5
C18—C21—H21B	109.5	C47—C55—H55B	109.5
H21A—C21—H21B	109.5	H55A—C55—H55B	109.5
C18—C21—H21C	109.5	C47—C55—H55C	109.5
H21A—C21—H21C	109.5	H55A—C55—H55C	109.5
H21B—C21—H21C	109.5	H55B—C55—H55C	109.5
O2—C22—C27	121.6 (3)	N3—C56—C46	113.9 (2)
O2—C22—C23	118.3 (3)	N3—C56—H56A	108.8
C27—C22—C23	120.1 (3)	C46—C56—H56A	108.8
C24—C23—C22	116.2 (3)	N3—C56—H56B	108.8
C24—C23—C28	123.7 (3)	C46—C56—H56B	108.8
C22—C23—C28	120.1 (3)	H56A—C56—H56B	107.7
C23—C24—C25	124.4 (3)	N3—C57—C58	112.3 (2)
C23—C24—H24	117.8	N3—C57—H57A	109.2
C25—C24—H24	117.8	C58—C57—H57A	109.2
C24—C25—C26	118.1 (3)	N3—C57—H57B	109.2
C24—C25—C35	120.1 (3)	C58—C57—H57B	109.2
C26—C25—C35	121.8 (3)	H57A—C57—H57B	107.9
C27—C26—C25	117.6 (3)	C59—C58—C57	109.6 (3)
C27—C26—C34	119.5 (3)	C59—C58—C62	112.7 (3)
C25—C26—C34	122.9 (3)	C57—C58—C62	110.5 (3)
C26—C27—C22	123.2 (3)	C59—C58—H58	108.0
C26—C27—H27	118.4	C57—C58—H58	108.0
C22—C27—H27	118.4	C62—C58—H58	108.0
C23—C28—C29	111.5 (3)	C60—C59—C58	112.2 (3)
C23—C28—C33	114.8 (3)	C60—C59—H59A	109.2
C29—C28—C33	109.4 (3)	C58—C59—H59A	109.2
C23—C28—H28	106.9	C60—C59—H59B	109.2
C29—C28—H28	106.9	C58—C59—H59B	109.2
C33—C28—H28	106.9	H59A—C59—H59B	107.9
C28—C29—C30	113.1 (3)	C59—C60—C63	113.2 (3)
C28—C29—H29A	108.9	C59—C60—C61	109.9 (3)
C30—C29—H29A	108.9	C63—C60—C61	109.9 (3)
C28—C29—H29B	108.9	C59—C60—H60	107.9
C30—C29—H29B	108.9	C63—C60—H60	107.9
H29A—C29—H29B	107.8	C61—C60—H60	107.9
C31—C30—C29	113.5 (4)	N3—C61—C60	112.5 (2)
C31—C30—H30A	108.9	N3—C61—H61A	109.1
C29—C30—H30A	108.9	C60—C61—H61A	109.1
C31—C30—H30B	108.9	N3—C61—H61B	109.1
C29—C30—H30B	108.9	C60—C61—H61B	109.1
H30A—C30—H30B	107.7	H61A—C61—H61B	107.8
C32—C31—C30	111.2 (4)	C58—C62—H62A	109.5
C32—C31—H31A	109.4	C58—C62—H62B	109.5
C30—C31—H31A	109.4	H62A—C62—H62B	109.5
C32—C31—H31B	109.4	C58—C62—H62C	109.5
C30—C31—H31B	109.4	H62A—C62—H62C	109.5
H31A—C31—H31B	108.0	H62B—C62—H62C	109.5
C31—C32—C33	111.4 (4)	C60—C63—H63A	109.5
C31—C32—H32A	109.3	C60—C63—H63B	109.5
C33—C32—H32A	109.3	H63A—C63—H63B	109.5
C31—C32—H32B	109.3	C60—C63—H63C	109.5
C33—C32—H32B	109.3	H63A—C63—H63C	109.5
H32A—C32—H32B	108.0	H63B—C63—H63C	109.5
			
O1—C1—C2—C3	173.8 (2)	C28—C29—C30—C31	−51.8 (6)
C6—C1—C2—C3	−7.2 (4)	C29—C30—C31—C32	52.4 (6)
O1—C1—C2—C7	−5.6 (4)	C30—C31—C32—C33	−55.0 (6)
C6—C1—C2—C7	173.4 (3)	C31—C32—C33—C28	57.5 (5)
C1—C2—C3—C4	2.7 (4)	C23—C28—C33—C32	179.1 (3)
C7—C2—C3—C4	−178.0 (3)	C29—C28—C33—C32	−54.8 (5)
C2—C3—C4—C5	4.0 (4)	C36—N2—C35—C25	71.5 (4)
C2—C3—C4—C14	−174.4 (3)	C40—N2—C35—C25	−167.8 (3)
C3—C4—C5—C6	−6.1 (4)	C24—C25—C35—N2	−92.3 (4)
C14—C4—C5—C6	172.3 (2)	C26—C25—C35—N2	85.6 (4)
C3—C4—C5—C13	172.5 (3)	C40—N2—C36—C37	60.0 (4)
C14—C4—C5—C13	−9.0 (4)	C35—N2—C36—C37	−179.1 (3)
C4—C5—C6—C1	1.7 (4)	N2—C36—C37—C38	−58.3 (4)
C13—C5—C6—C1	−177.0 (2)	N2—C36—C37—C41	177.0 (4)
O1—C1—C6—C5	−175.8 (2)	C36—C37—C38—C39	53.4 (4)
C2—C1—C6—C5	5.3 (4)	C41—C37—C38—C39	177.4 (4)
C3—C2—C7—C8	100.7 (4)	C37—C38—C39—C40	−51.1 (5)
C1—C2—C7—C8	−79.9 (4)	C37—C38—C39—C42	−175.4 (4)
C3—C2—C7—C12	−24.4 (5)	C36—N2—C40—C39	−58.8 (4)
C1—C2—C7—C12	155.0 (4)	C35—N2—C40—C39	179.2 (3)
C12—C7—C8—C9	−51.9 (6)	C38—C39—C40—N2	54.1 (4)
C2—C7—C8—C9	178.7 (4)	C42—C39—C40—N2	179.6 (3)
C7—C8—C9—C10	49.2 (8)	O3—C43—C44—C45	−176.3 (2)
C8—C9—C10—C11	−45.8 (9)	C48—C43—C44—C45	4.2 (4)
C9—C10—C11—C12	47.8 (8)	O3—C43—C44—C49	4.8 (4)
C8—C7—C12—C11	55.9 (6)	C48—C43—C44—C49	−174.7 (2)
C2—C7—C12—C11	−176.0 (4)	C43—C44—C45—C46	0.5 (4)
C10—C11—C12—C7	−54.8 (7)	C49—C44—C45—C46	179.4 (2)
C15—N1—C14—C4	−74.1 (3)	C44—C45—C46—C47	−4.9 (4)
C19—N1—C14—C4	164.9 (2)	C44—C45—C46—C56	175.1 (2)
C3—C4—C14—N1	96.3 (3)	C45—C46—C47—C48	4.6 (4)
C5—C4—C14—N1	−82.1 (3)	C56—C46—C47—C48	−175.4 (2)
C19—N1—C15—C16	−60.2 (3)	C45—C46—C47—C55	−176.1 (2)
C14—N1—C15—C16	178.7 (2)	C56—C46—C47—C55	3.9 (4)
N1—C15—C16—C17	56.6 (3)	C46—C47—C48—C43	−0.1 (4)
N1—C15—C16—C20	−178.9 (2)	C55—C47—C48—C43	−179.5 (2)
C20—C16—C17—C18	−175.8 (3)	O3—C43—C48—C47	176.0 (2)
C15—C16—C17—C18	−51.9 (3)	C44—C43—C48—C47	−4.5 (4)
C16—C17—C18—C19	51.5 (3)	C45—C44—C49—C54	−95.5 (3)
C16—C17—C18—C21	174.7 (3)	C43—C44—C49—C54	83.3 (3)
C15—N1—C19—C18	59.1 (3)	C45—C44—C49—C50	28.6 (4)
C14—N1—C19—C18	−179.2 (2)	C43—C44—C49—C50	−152.6 (3)
C17—C18—C19—N1	−55.0 (3)	C44—C49—C50—C51	179.0 (3)
C21—C18—C19—N1	−178.8 (3)	C54—C49—C50—C51	−56.6 (4)
O2—C22—C23—C24	−176.5 (3)	C49—C50—C51—C52	56.0 (5)
C27—C22—C23—C24	3.8 (4)	C50—C51—C52—C53	−54.2 (5)
O2—C22—C23—C28	2.9 (4)	C51—C52—C53—C54	54.2 (5)
C27—C22—C23—C28	−176.7 (3)	C44—C49—C54—C53	−176.8 (3)
C22—C23—C24—C25	1.4 (5)	C50—C49—C54—C53	56.2 (4)
C28—C23—C24—C25	−178.0 (3)	C52—C53—C54—C49	−55.7 (5)
C23—C24—C25—C26	−5.3 (5)	C57—N3—C56—C46	73.1 (3)
C23—C24—C25—C35	172.7 (3)	C61—N3—C56—C46	−166.0 (3)
C24—C25—C26—C27	3.8 (4)	C45—C46—C56—N3	−92.0 (3)
C35—C25—C26—C27	−174.2 (3)	C47—C46—C56—N3	88.0 (3)
C24—C25—C26—C34	−177.0 (3)	C61—N3—C57—C58	59.3 (3)
C35—C25—C26—C34	5.0 (4)	C56—N3—C57—C58	179.7 (2)
C25—C26—C27—C22	1.3 (4)	N3—C57—C58—C59	−56.7 (4)
C34—C26—C27—C22	−177.9 (3)	N3—C57—C58—C62	178.5 (3)
O2—C22—C27—C26	175.1 (3)	C57—C58—C59—C60	53.2 (4)
C23—C22—C27—C26	−5.3 (4)	C62—C58—C59—C60	176.7 (3)
C24—C23—C28—C29	−92.1 (4)	C58—C59—C60—C63	−175.8 (3)
C22—C23—C28—C29	88.5 (4)	C58—C59—C60—C61	−52.5 (4)
C24—C23—C28—C33	33.0 (5)	C57—N3—C61—C60	−58.6 (3)
C22—C23—C28—C33	−146.4 (3)	C56—N3—C61—C60	179.7 (3)
C23—C28—C29—C30	179.8 (3)	C59—C60—C61—N3	55.3 (4)
C33—C28—C29—C30	51.7 (5)	C63—C60—C61—N3	−179.5 (3)

### Hydrogen-bond geometry (Å, °)

**Table:**

*D*—H···*A*	*D*—H	H···*A*	*D*···*A*	*D*—H···*A*
O1—H1···N1^i^	0.82	2.03	2.843 (3)	170
C13—H13B···O1^i^	0.96	2.52	3.335 (4)	143
O2—H2···N2^ii^	0.82	2.03	2.845 (3)	171
C34—H34C···O2^ii^	0.96	2.49	3.348 (4)	149
O3—H3···N3^iii^	0.82	2.02	2.831 (3)	174
C55—H55C···O3^iii^	0.96	2.58	3.404 (4)	145

Symmetry codes: (i) −*x*+2, −*y*+1, −*z*+1; (ii) −*x*+1, −*y*+1, −*z*; (iii) −*x*, −*y*, −*z*+1.
